# Nutritional Exchanges Within Tumor Microenvironment: Impact for Cancer Aggressiveness

**DOI:** 10.3389/fonc.2020.00396

**Published:** 2020-03-24

**Authors:** Giuseppina Comito, Luigi Ippolito, Paola Chiarugi, Paolo Cirri

**Affiliations:** ^1^Department of Experimental and Clinical Biomedical Sciences, University of Florence, Florence, Italy; ^2^Excellence Center for Research, Transfer and High Education DenoTHE, University of Florence, Florence, Italy

**Keywords:** tumor microenvironment, OXPHOS, EVs, EMT, CAFs

## Abstract

Neoplastic tissues are composed not only by tumor cells but also by several non-transformed stromal cells, such as cancer-associated fibroblasts, endothelial and immune cells, that actively participate to tumor progression. Starting from the very beginning of carcinogenesis, tumor cells, through the release of paracrine soluble factors and vesicles, i.e., exosomes, modify the behavior of the neighboring cells, so that they can give efficient support for cancer cell proliferation and spreading. A mandatory role in tumor progression has been recently acknowledged to metabolic deregulation. Beside undergoing a metabolic reprogramming coherent to their high proliferation rate, tumor cells also rewire the metabolic assets of their stromal cells, educating them to serve as nutrient donors. Hence, an alteration in the composition and in the flow rate of many nutrients within tumor microenvironment has been associated with malignancy progression. This review is focused on metabolic remodeling of the different cell populations within tumor microenvironment, dealing with reciprocal re-education through the symbiotic sharing of metabolites, behaving both as nutrients and as transcriptional regulators, describing their impact on tumor growth and metastasis.

## Introduction

### Tumor Microenvironment

A solid tumor is a dysfunctional neoplastic tissue characterized by uncontrolled growth and chaotic histological organization and it is composed, in addition to cancer cells, by heterogeneous subsets of non-transformed cells, such as mesenchymal stem cells, fibroblasts, endothelial cells, adipocytes and immune cells, establishing a complex tumor microenvironment (TME) with peculiar structural and biophysical characteristics [i.e., altered extracellular matrix (ECM) composition, acidity and hypoxia]. The features of the neoplastic parenchyma are well instructed through a complex interplay between cancer and stromal cells, orchestrated by soluble factors, metabolites, extracellular vesicles (EVs), as well as cell-to-cell interaction.

In physiologic conditions fibroblasts are the main cellular component of connective tissue and they are involved in providing structural scaffolding and trophic ancillary function for the epithelial cells of the tissues (1). In tumors, cytokines released by cancer cells convert fibroblasts into a permanently activated, myofibroblast-like, form called cancer associated fibroblasts (CAFs) (2). This chronic activation of fibroblasts within TME is crucial for cancer progression. Indeed, CAFs are responsible for an abnormal ECM deposition and remodeling, for a persistent inflammation mediated by soluble factors (i.e., cytokines) (SDF-1, CXCL14, etc.) leading to new vessels formation and recruitment of immune cells within the TME, events particularly important for the nutrients supply and metastatic dissemination, respectively (3). CAFs also exert an immunomodulating role, mainly by enhancing the M2/M1 macrophage and the Th2/Th1 ratio (4, 5). Besides their immunomodulating and pro-angiogenic activity, CAFs are able to promote epithelial–mesenchymal transition (EMT) in cancer cells, conferring them proinvasive and stem-like features (6). Finally, CAFs play a mandatory role in cancer cells dissemination, since they can escort metastatic cancer cells in the bloodstream, favoring their implantation at distal sites (7).

Mesenchymal stem cells (MSCs) are multipotent stromal cells recruited into TME mainly from adipose tissue and bone marrow in response to several growth factors, i.e., platelet derived growth factor (PDGF), vascular endothelial growth factor (VEGF), transforming growth factor-β (TGF-β) as well as EVs released by cancer cells (8). MSCs possess self-renewal ability and are able to differentiate into several cell types within TME, such as CAFs. For example, in neuroblastoma tumor CAFs share phenotypic and functional characteristics with bone marrow-derived MSCs (9), while *in vitro* conditioning of bone marrow-derived MSCs cells with tumor-derived medium lead to the acquiring of a CAF-like phenotype sustaining tumor growth both *in vitro* and *in vivo* (10). In addition to the pro-tumorigenic functions, broadly shared with CAFs, MSCs show an immunosuppressive role in both prostate and melanoma cancer models (11, 12).

Neo-angiogenesis, the growth of new blood vessels from the existing vasculature, is a key step in tumor progression. Under the stimulation of pro-angiogenetic cytokines, endothelial cells within TME provokes a wide but disorganized rearrangement of vessel architecture characterized by altered permeability which is crucial for tumor cells metastatic spreading. In addition, tumor endothelial cells can secrete angiocrine factors, such as CSF-1 or interleukin (IL)-8 (13) promoting cancer cells migration along with neutrophils infiltration, hence widening their functions in tumor progression (14).

Tumor- or CAF-derived cytokines also are able to induce monocytes recruitment within the tumor mass where they were activated to M1-like macrophages by CSF-1 and IFN-γ (15). Conversely, macrophages stimulation with type 2 T helper cell cytokines, such as IL-4 and IL-10, leads to phenotype called M2-like endowed with pro-tumor characteristics, likely taking part in all steps of the metastatic route (16).

Tumor-associated neutrophils (TANs) are divided into two sub-populations, showing antitumoral activity (N1-like phenotype) or protumoral activity (N2 phenotype). Neutrophils, recruited in TME by CXCL2 and CXCL5 cytokines, actively participate in the metastatic process by enhancing tumor cell expression of pro-metastatic genes (15), as well as associating with circulating breast tumor cells, helping them to proliferate once they reach the secondary site (11).

Many lymphocytes subtypes are present in TME as CD4^+^ helper cells, immunosuppressive regulatory T-cells (Tregs) and CD8^+^ cytotoxic T-cells, recruited by several chemokines produced by cancer and stromal cells. The histological origin, the composition and the density of the cells that constitute tumor-infiltrating lymphocytes, together with hormonal context within TME can determine tumor progression and clinical outcome. For example, a lot of evidence have addressed the role of cytotoxic CD8^+^ T cells, whose presence and activity is associated with a good prognosis, while the infiltration of Tregs, an immunosuppressive T cell subpopulation, has been shown to be associated with poor prognosis in several tumors (17).

Adipocytes are recently emerging as important contributors to cancer progression. Cancer cells through chemokines secretion can convert adipocytes into their activated form Cancer-Associated Adipocytes (CAAs), that has been reported to promote IL-6-mediated EMT in cancer cells (18). CAAs-secreted leptin has a proliferative effect on cancer cells (19) as well as a pro-angiogenic role (20). On the contrary, adiponectin secretion decreases in CAA with respect to normal adipocytes, suggesting an anti-proliferative effect of this adipokine on cancer cells (19).

Recent advances in tumor biology showed the importance of a highly tuned exchange of nutrients within TME, impacting on tumor progression (21). A consequence of the cytokines-mediated cross-talk between cancer and stromal cells is the metabolic reprogramming of all cellular components of the tumor, aimed at maximizing the proliferative capacity of tumor cells. In this view CAFs, which are the major component of tumor stroma, together with adipocytes, give a feed support to tumor cells, increasing their growth rate. In addition, some nutrients exchanged in the TME, also play an essential signaling function acting as epigenetic switches, leading to activation of EMT and inhibition of immune cell response. The multifaceted significance of nutrients exchange is discussed in the chapters below.

### Metabolic Deregulation in Cancer

A tumor, consisting of a heterogeneous and complex network of cancer and stromal cell populations, needs to adapt all the metabolic functions to support the demands of uncontrolled growth and to support disease progression. The metabolic alterations of a tumor come from both the oncogenic signaling that orchestrate distinct metabolic pathways and the environmental context that promotes nutrient-based intercellular cross-talk and/or competition.

Actually, it is widely recognized that cancer cells need to meet their bioenergetic and biosynthetic demands to maintain a high tumor cell growth rate. Tumor cells require a high rate of biosynthesis of macromolecules (lipids, amino acids, nucleic acids) in order to maintain the cellular redox balance and, at the same time, to compensate their energy-consuming processes, ultimately culminating in fueling tumor growth and progression. However, the metabolic reprogramming of cancer cells is crucial also for the signaling role exerted by the metabolites.

Intriguingly, the metabolic flux is mainly derived from the glucose in cancer cells, known as the Warburg effect, that is the ability of cancer cell to massively upload glucose, thanks to the upregulation of glucose transporters GLUT1-3, in order to (i) provide precursors and intermediary metabolites, useful for the tumor-associated biosynthetic machinery, and to (ii) produce high amounts of lactate, even in the presence of oxygen. Warburg metabolism is one of the most commonly observed examples of metabolic reprogramming in highly proliferating cells, such as cancer cells and non-transformed cells (i.e., T lymphocytes), taking advantage from the rapid production of ATP and the synthesis of glucose-derived macromolecules (22).

The collateral metabolic fluxes arising from aerobic glycolysis lead to the activation of specific pathways such as the pentose-phosphate-pathway (PPP) and the one-carbon metabolism. PPP is important for tumor cells as it generates pentoses useful for DNA/RNA synthesis and feeds the nicotinamide-adenine dinucleotide phosphate (NADPH) pool, which is needed for fatty acid synthesis and cell survival under oxidative stress conditions. The harsh TME, as well the oncogenic background, are responsible of the increase of reactive oxygen species (ROS) in tumor cells. These highly reactive molecules can detrimentally modify the intracellular environment as well as activate certain pro-tumoral signaling pathways, under certain sub-toxic levels. Accordingly, to challenge the toxic levels of ROS, tumor cells increase their antioxidant capacity to allow cancer progression and PPP activation is oriented in such way. Oxidative stress can be counteracted by the production of NADPH by the oxidative branch of the pentose phosphate pathway, as it is used by the glutathione reductase enzyme in the reduction reaction of oxidized glutathione (GSSG). To note, glutathione (GSH) is the one of most important antioxidant molecule within the cell, it is synthesized from glutamine carbons and conditions of oxidative stress increase the conversion of GSH (reduced, physiological form) to GSSG (oxidized), which is potentially toxic for the cell, as it acts as a pro-oxidant (23). The deregulation of glutathione metabolism is broadly identifiable in the majority of cancers as the genes involved in GSH turnover or utilization are under the transcriptional control of classical tumorigenic pathways, primarily the nuclear factor erythroid 2-related factor 2 (NRF2) signaling which drives the antioxidant response and control the transcription of glutamate-cysteine ligase, the first enzyme of the cellular GSH biosynthetic pathway. In addition, the hypoxic signaling is a driving force for the activation of GSH production and it has been associated with the enrichment of breast cancer stem cell niche following chemotherapy treatments (24). GSH alterations have been identified in metabolically deregulated tumors, such as tumors deficient for fumarate hydratase enzyme. Strikingly, the accumulation of fumarate in FH-deficient cancer cell lines leads to the formation of a peculiar molecule between fumarate and glutathione (GSH), which depletes intracellular NADPH and enhances oxidative stress (25). Also, in MYC-driven liver tumors, particular for a decreased incorporation of glutamine, the attenuation of expression of glutamate-cysteine ligase contributes to GSH depletion (26). A key role of glutathione is also emerging in the context of tumor microenvironment. In particular, CAFs were shown to diminish the accumulation of genotoxic agents in cancer cells in a glutathione-dependent manner. In fact, CAFs release high levels of thiols, including glutathione and cysteine, which increase intracellular GSH levels in tumors counteracting drug-dependent oxidative stress and apoptotic response (27, 28). Furthermore, glycolysis can divert glucose-derived intermediates to one-carbon pathway that is important for serine synthesis (29). It supplies methyl groups to the one-carbon and folate pools, contributing to amino acid and nucleotides synthesis, methylation reactions, and NADPH production. Finally, the Warburg-associated fermentation of pyruvate into lactate, catalyzed by lactate dehydrogenase A enzyme, culminates in its extrusion in the extracellular milieu via the monocarboxylic acids transporter MCT4. Lactate release, coupled with H^+^, increases external acidity and deliver to TME a peculiar molecule losing its classification as waste product, as it plays both a signaling and a metabolic role, thereby altering the immune cell landscape, increasing tumor invasive capacity and supplying an appealing carbon source for other cell populations (30) (see below).

Warburg metabolism is an aspect of a highly multilayered cancer metabolism, as cancer cells have adapted multiple mechanisms to exploit metabolic substrates through mitochondria. To note, many reactions of the tricarboxylic acid (TCA) cycle are reversible and multiple mitochondria-associated anaplerotic circuitries ensure such a metabolic adaptation of cancer cells.

In addition to glucose-derived pyruvate, fatty acids (FAs) and amino acids can feed the TCA cycle to sustain mitochondrial activity in malignant cells and produce ATP via oxidative phosphorylation. A key role of the TCA cycle in proliferating cells is to act as a biosynthesis hub and this function differs from that occurring in non-proliferating cells, where TCA cycle serves to provide the maximal ATP production. During tumor cell proliferation, however, much of the carbon that enters the TCA cycle is used in biosynthetic pathways. In this scenario, tumor mitochondrial metabolism represents a cataplerotic center by providing building blocks for anabolic processes. Synthesis of lipids (fatty acids, cholesterol, and isoprenoids) is a crucial example of cataplerosis in tumor cells and the activation of lipid biogenesis could play an active role in cell transformation and cancer development, as lipids have important roles in membrane structure, cellular signaling and protein regulation, beyond energetics. Glucose is a major lipogenic substrate as it can be oxidized and mediates the transfer of mitochondrial citrate out to the cytosol to be converted to oxaloacetate (OAA) and the lipogenic precursor acetyl-CoA, which can either be used for fatty acid and cholesterol synthesis or for epigenetic purposes (i.e., acetylation reactions), by providing a pool for chromatin-modifying enzymes such as the acetyltransferases (31). However, the biosynthesis of fatty acid *chains*, upon conversion of citrate to acetyl-CoA via ATP-citrate lyase (ACLY), is sustained by the carboxylation of cytosolic acetyl-CoA by acetyl-CoA carboxylase (ACC) to produce malonyl-CoA, which is further assembled into long fatty acid chains by fatty acid synthase (FASN). As ACLY, ACC, and FASN are frequently upregulated in tumor cells and their inhibition reduces tumor growth, it is widely recognized that the increased capacity for producing lipids *de novo* is a crucial determinant for the tumor progression. In addition, cholesterol synthesis plays a role in the tumor malignancy, as the interference with such pathway through statins treatment provokes a detrimental effect on tumor growth *in vitro* and *in vivo* (32).

The major anaplerotic substrate in growing cells is glutamine, the most rapidly consumed nutrient by many human cancer cells. Indeed, most of them display addiction to glutamine, thereby boosting its uptake mainly through SLC1A5/ASCT2 transporter, and its catabolism (glutaminolysis) via the activity of mitochondrial glutaminolytic enzymes, glutaminase and glutamate dehydrogenase. Glutamine entry and metabolism is mainly supported by c-Myc, a transcription factor upregulated in several cancer cells. To this end, c-Myc induces the transcription of glutamine transporters, and of glutamine-utilizing enzymes, such as glutaminase, phosphoribosyl pyrophosphate synthetase and carbamoyl-phosphate synthetase 2.

Glutamine is important for energetic demands of cancer cells as it provides carbons to replenish the TCA cycle. However, it also provides nitrogen for biosynthesis of purine and pyrimidine nucleotides, as well as of nonessential amino acids. Glutamine metabolism also contributes to the production of glutathione, thus playing a role in the cellular anti-oxidant defense, and serves as a precursor to lipid synthesis via α-ketoglutarate (KG)-to-citrate conversion namely reductive carboxylation (33, 34). In addition, many epigenetic modifications and cellular processes are regulated by glutamine-derived α-KG, which is a cofactor of dioxygenase enzymes, including the ten eleven translocases (TET) family and the Jumonji (JMJ) family, thereby affecting, respectively, DNA and histone demethylation (35).

In keeping, in addition to catabolic, energetic and anabolic requirements for cancer growth by exploiting TCA cycle, an intracellular signal transduction cascade is mediated by other TCA cycle metabolites (36, 37). In tumors harboring the loss of the mitochondrial enzymes succinate dehydrogenase or fumarate hydratase, the respective accumulation of succinate or fumarate has been shown to inhibit the enzymatic activity of α-KG-dependent dioxygenases. Hence, these enzymes are important for different purposes such as hypoxia inducible factor (HIF)-1 stability, as well as epigenome rewiring. In keeping, in tumors that have lost succinate dehydrogenase or fumarate hydratase, HIF-1 is activated under normoxic conditions, resulting in the activation of pseudohypoxic pathways and in the enhancement of tumor malignancy (38).

Thus, beyond the genetic alterations, the ability of tumor cells to engage different metabolic behaviors according to the metabolic scenario provided by the microenvironment (oxygen levels, austere availability of nutrients, stromal cues) greatly contributes to a high metabolic plasticity which consequently increases tumor heterogeneity. Indeed, a tumor cell needs to face the environmental scenario, by displaying a metabolic plasticity useful to orchestrate the selection, the upload and the consequent exploitation of the available nutrients in the TME. Thus, the metabolic reprogramming occurring in a cancer cell encompasses multiple strategies, among which is a non-cell autonomous one, mainly involving the tumor-associated stromal components that supply nutrients and establish metabolic networks with tumor cell compartment, thereby shaping their malignant phenotype.

### Nutrients Exchanged in TME

**Figure 1:**
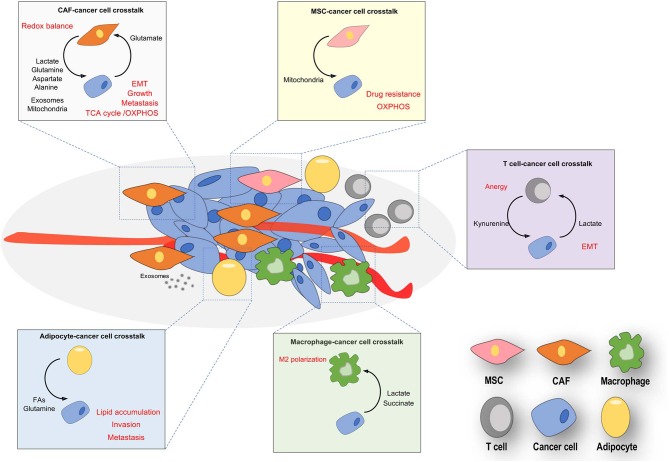
Nutrients-based cross-talks between stromal and cancer cells in TME. Resident and recruited stromal cells (CAFs, endothelial, adipocytes, T cells and macrophages) often are phenotypically conditioned by nutrients released by cancer cells, thereby provoking a both reactive and immunosuppressive environment. Cancer cells are educated by stromal nutrients to undergo a metabolic and phenotypic reprogramming, both crucial for the cancer metastasis and progression. The main exchanged nutrients (in bold) promote reciprocal phenotypic effects (in red) in stromal and tumor compartments.

Besides the metabolic reprogramming of a tumor cell harboring high mutational landscape, recent discoveries have highlighted nutrients available in the TME as crucial molecules acting on the acquisition of a peculiar metabolic and phenotypic plasticity in tumor cells allowing them to adapt to the peculiar features of the TME they face with (e.g., cytokine delivery, oxidative, acidic, and nutritional stress). Tumor-associated stromal and tumor populations dynamically communicate each other through metabolic connections, causing a reciprocal tumor-stroma metabolic interplay. Such metabolic symbiosis reasonably have the advantage to supply each other different metabolites able to reprogram anabolic and catabolic processes in the recipient subpopulations (Figure 1). Of note, nutrients arisen by stromal populations can overcome metabolic constrains within the tumor, circumventing oncogenes or tumor suppressors regulation of several metabolic enzymes, thus rewriting cancer mass evolution.

Several nutrients will be exchanged within TME, as explained below.

#### Lactate

Mitochondrial exploitation of lactate over glucose has been reported in human lung tumors, highlighting the contribution of stroma for anabolic purposes and TCA replenishment driven by such metabolite (39). A clear lactate-based tumor-stroma cross-talk has been reported in several tumor models, including the prostate carcinoma. CAFs predominantly exhibit aerobic glycolysis and secrete lactate through monocarboxylate transporter (MCT)-4, whose expression is under the redox or succinate-dependent HIF-1 control (40, 41). Cancer cells educate CAFs to secrete lactate, exploiting directly the environmental lactate, uploaded through MCT-1. Once imported, lactate is able to rewire cancer cell metabolism, causing a shift from glycolysis to oxidative phosphorylation (OXPHOS) (42). The inward of stromal lactate provokes the unbalance of NAD^+^/NADH ratio (see the lactate-to-pyruvate conversion and its oxidation), causing *ad hoc* adaptive changes in cancer cells, such as sirtuin1-mediated de-acetylation/activation of the transcriptional co-activator peroxisome proliferator-activated receptor-gamma coactivator-1 (PGC-1α). This molecular signature has been found as crucial for the enhancement of the tumor mitochondrial mass and function of stroma-reprogrammed prostate cancer cells, as reported in other models of tumor progression (43, 44). Moreover, the simultaneous increase of the GLUT-1 carrier in CAFs, as well as activation of the mitochondrial pyruvate dehydrogenase complex, concur to significantly reprogram the metabolism of both tumor and stromal compartments establishing a metabolic symbiosis (45, 46). The *in vitro* definition of such metabolic symbiosis has been confirmed *in vivo* by recent isotope tracer measurements, showing a rapid exchange of lactate between the tumor and circulation in several cancer models (39, 47). Interestingly, the MCT-mediating lactate influx and efflux activity involves protons (H^+^), thereby leading to an apparent paradox. As several accessory cells in TME concur to decrease extracellular pH [due to the overexpression of carbonic anhydrases or proton pumps, commonly occurring in both cancer cells and CAFs (48, 49)], lactate/H^+^-coupled transport by MCTs tends to drive lactate from the interstitium into tumor cells (50), allowing them to anabolize this nutrient (51). Furthermore, the physical association between carbonic anhydrases (CA) and lactate transporters MCT-1/4 plays a key role in the regulation of the directional flux of lactate, as well as in the protonation/deprotonation of proteins, thereby affecting their functions. CAII is associated to the cytosolic part of the MCT transporters, while CAIX is associated to the extracellular face and MCT-assembled CD147 chaperone. MCT-associated CAs non-enzymatically cooperate to drive the lactate/H^+^ symport/export in/by cancer cells. Indeed, protons diffuse very slowly within the cell (52); for this reason, in order to allow a more efficient extrusion of H^+^ and lactate from the cell, the MCT does not extract protons directly from cytosol, but rather from protonated residues located in a peculiar antenna of CAII. Similarly to the cytoplasm, the diffusion of H^+^ in the extracellular space is restricted and protons have to be removed from the extracellular side by CAIX, shuttled to protonated residues and then released to the extracellular space. Hence, lactate bi-directional flux is strictly linked to acidity, both intracellular and extracellular, and mostly to CAs activity. Fascinatingly, as these enzymes links acidity to 1-C metabolism, catalyzing the formation of both H^+^ and ${\text{HCO}}_{3}^{-}$, it is conceivable that lactate flux leads to regulation of one carbon metabolism as well. Importantly, besides its role in the modulation of MCT-mediated lactate transport into cancer cells, the high amount of H^+^ within the TME can modify other aspects of tumor metabolism, as it promotes a preferential exploitation of glutamine and lipids—as sources of energy and biosynthesis—in cancer cells, over the canonical glycolytic metabolism. Indeed, a decrease in HIF1α activation (resulting from direct acetylation) concomitantly with a reduction in the expression of glycolytic enzymes as well as the glucose transporter GLUT1 and the lactate transporter MCT4 has been reported (53, 54). Also, acidosis drives the reprogramming of lipid metabolism by triggering an increase in HIF2α activity which stimulates the reductive and oxidative glutamine metabolism, ultimately sustaining the co-existence of synthesis and oxidation of Fas (55). Notably, the newly synthetized lipids, stored in lipid droplets, represent a readily available energy to support *anoikis* resistance and invasiveness in cancer cells adapted to the acidic conditions (56).

Finally, immune cells may be forced to experience environmental lactate with beneficial or detrimental consequences on their differentiation. Indeed, in a prostate cancer model, lactate released by glycolytic CAFs causes a clear shaping of T-cell polarization, by reducing the percentage of the anti-tumoral Th1 subset cells and increasing pro-tumorigenic Treg cells subpopulation. Both Th1 and Treg cells are reprogrammed by an activation of a lactate-driven epigenetic pathway, causing activation of T-bet or NF-kB/FoxP3 transcription factors. This lactate-based reprogramming of T-cell response leads to enhance malignancy of prostate tumors, thereby confirming the immunomodulatory role of lactate (51). Furthermore, cancer cell-derived lactate is able to polarize M1 macrophages into M2 ones, activating different signaling cascades (i.e., VEGF, arginase-1) in macrophages undergoing pro-tumor differentiation (57). Notably, another signaling role of the environmental lactate has been investigated in endothelium as lactate could be up-taken by endothelial cells through the MCT-1, thus stimulating the autocrine NF-κB/IL-8 (CXCL8) pathway which affects tumor angiogenesis in terms of endothelial migration, vessels permeability and morphology (58). Importantly, besides the canonical role as nutrient, lactate has been shown to act as a hormone, as it is able to activate signaling pathways downstream the hydroxycarboxylic acid receptor 1, formerly known as G protein-coupled receptor 81 (GPR81). This receptor, sensitive to low concentrations of lactate (1–5 mM), is coupled to G_i/q_, and activation of the receptor results in decreased cellular levels of cAMP and increased cellular levels of Ca^2+^, leading a fascinating hypothesis of an autocrine and paracrine role of lactate in cancerous and stromal cell types via surface receptor (59, 60). Very recently, another non-nutritional role of lactate has been reported by Zhang et al. (61). These authors reported that lactate produced in hypoxic environment is involved in a peculiar post-transcriptional modification of histones (i.e., lactylation), a process occurring with different temporal dynamics from acetylation and, so far, involved in wound healing and pro-inflammatory signals (61). Although the role of lactylation in TME deserves future considerations, it is likely that this hypothesis deserves consideration in future studies. However, the lack of definitive information regarding the microenvironmental fluxes of lactate in the different cellular compartments highlights the general need for enlarging and setting new tracing studies in the tumor extracellular milieu.

#### TCA Intermediates

Oncometabolites are a group of metabolites, including succinate, fumarate, and 2-hydroxyglutarate, accumulated in cancer cells generally as a consequence of mutations in genes coding for the related metabolic enzymes, that are succinate dehydrogenase (SDH), fumarate hydratase, or isocitrate dehydrogenase, or of alterations in their enzymatic activity (62). These metabolites are involved in the dysregulation of several cellular processes, mainly through the competitive inhibition of α-KG-dependent dioxygenases, causing pseudohypoxia via HIF-1 stabilization, protein post-transcriptional modifications, as well as epigenetic alterations in cancer cells. Mutations of SDH subunit genes are recurrent in some cancer types including hereditary pheochromocytoma syndrome and paraganglioma. In any case, there is a loss of function of the SDH enzyme, causing succinate accumulation (63, 64). Germline mutations in the SDH subunits have also been shown to cause gastrointestinal renal, pancreatic neuroendocrine, thyroid, and neuroblastoma tumors (65), although SDH activity can also be epigenetically inhibited via the binding of the chaperone tumor necrosis factor receptor-associated protein 1 (49), or through the competitive inhibition of the metabolite itaconate highly enriched in reactive macrophages (66). Increased concentrations of succinate may induce metabolic reprogramming within TME and concur to promote cancer progression. Accumulation of succinate is correlated with a state of pseudohypoxia, due to its ability to inhibit prolyl hydroxylation of HIF-1α, leading to stabilization of the transcription factor and activation of HIF-controlled genes involved in glycolysis, angiogenesis, and EMT (67). Moreover, accumulation of fumarate can reduce the expression of the anti-metastatic miRNA cluster mir-200ba429, by inhibiting demethylation of the CpG islands in the regulatory region via regulation of DNA demethylases TETs. This epigenetic rewiring promotes activation of EMT programme and the increase of metastatic potential in a model of renal cancer (38).

Moreover, succinate post-translationally modifies lysine residues of proteins through succinylation, including L-lactate dehydrogenase A, glyceraldehyde 3-phosphate dehydrogenase, glutamate carrier-1, uncoupling protein-1 and malate dehydrogenase, all enzymes involved in the reprogramming of cancer cell metabolism (68, 69).

Beside the direct role of oncometabolites in those cancer cells undergoing their accumulation, a new original view also supports the oncometabolites as signaling molecules, secreted by dying cells or by neighboring stromal populations. In this line, we have recently reported that exposure of prostate cancer cells to CAFs, while undergoing mitochondrial deregulation and OXPHOS addiction, leads to the accumulation of TCA cycle intermediates, consistently with lactate oxidative exploitation (51). Cancer cells, uploading lactate secreted by CAFs, fuel TCA cycle and accumulate succinate and fumarate, likely linked to their ability to drive a pseudohypoxic HIF-1-mediated EMT motility (51). Upon deregulation of TCA, succinate and fumarate are also secreted in TME, although indications on their specific destiny are lacking. In keeping with a role as extracellular signal, succinate can bind to its cognate receptor namely SUCNR1 (68). SUCNR1, belonging to the family of G protein-coupled receptors, is expressed in kidney, liver, brain, bone marrow, as well as in several cancers (70), and is reported to control cell proliferation, migration, capillary formation and development of new vessels formation, VEGF secretion, as well as stem cell functions (71, 72).

Cancer cells can either accumulate succinate, or eventually upload it from the TME. Indeed, a plasma membrane Na(^+^)-dependent dicarboxylic acid transporter NaDC3 (also called SLC13A3), able to specifically upload succinate, has been reported in prostate cancer cells. The real contribution of extracellular succinate is not clear, as the block of the succinate plasma membrane carrier is not sufficient to inhibit cancer growth in a PTEN-loss model of prostate cancer, while succinate-supported respiration is mandatory for prostate cancer malignancy (73). During inflammation, succinate may be secreted by inflammatory macrophages and accumulate into TME (74), as reported in murine ischemic tissues (75), central nervous system inflammation and in rheumatoid arthritis inflammation. Interestingly, macrophages express GPR91 and, in response to inflammatory signals like lipopolysaccaride, activate a GPR91-mediated signal transduction that sustains the pro-inflammatory phenotype and leads to IL-1β production (76). This represents a novel mechanism by which succinate fuels inflammation in an autocrine manner to sustain and amplify the inflammatory response (77).

However, fascinating evidence report that cancer cell-secreted succinate elicits M2 macrophage polarization and positively regulates cancer metastasis via SUCNR1 (78), thus enlarging the class of tumor metabolic factors affecting TME and tumor phenotypic rearrangement.

#### Citrate

Citrate is the primary substrate for fatty acid synthesis and is metabolized in the cytoplasm by ATP-citrate lyase to serve acetyl-CoA moieties for lipid synthesis. Citrate-derived acetyl-CoA also contributes to amino acid synthesis, as well as to protein acetylation (79, 80), both processes critical for proliferating cells. Sources of citrate for cancer cells are their own Krebs cycle, reductive carboxylation of α-KG originating from glutaminolysis (81), as well as the direct importation from TME through a plasma membrane-specific variant of the mitochondrial citrate transporter (82). Consistent with the hypothesis of extracellular citrate as a key nutrient able to affect cancer aggressiveness the blocking of the plasma membrane citrate carrier (variant of the SLC25A1), expressed in several malignant cancers, results in decreased tumor growth in immunodeficient mice and altered tumor metabolism. Moreover, decreased blood citrate levels have been associated with some tumors including those in the lung, bladder, and pancreas (83).

Finally, citrate, upon conversion into isocitrate, can also fuel itaconate biosynthesis as a TCA cycle by-product from the decarboxylation of *cis*-aconitate. Itaconate production is active in macrophages upon exposure to inflammatory stimuli, playing a direct antimicrobial effect, markedly affecting immunomodulation, suppression of inflammation and tolerance (66). Itaconate acts mainly by inhibiting SDH, causing accumulation of succinate in LPS activated macrophages, and this was associated to reduced mitochondrial respiration, ROS production, HIF-1 pseudohypoxic activation, pro-inflammatory cytokine release, and inflammasome activation (84). Although itaconate plays clearly a key role within TME by regulating macrophage activation, its release in TME has not been yet reported.

#### Glutamine and Other Aminoacids

Intriguingly, although lactate is the most abundant nutrient provided in the TME, CAFs are also able to supply amino acids like glutamine to cancer cells. Epithelial cancer cells incorporate fibroblasts-derived glutamine replenishing their TCA cycle, as well as promoting an increase in aspartate-mediated nucleotide anabolism, the accumulation of oxidized glutathione and the activation of protein synthesis (85). Glutamine dependency as it is exploited as a carbon source for the energetic purposes and as a nitrogen source for nucleotide biosynthesis reflects the fact such amino acid is the most commonly depleted amino acid in TME (33). In agreement, glutamine-restricted TME are truly dependent on tumor-stroma glutamine cross-feeding. In ovarian carcinoma, CAFs metabolism diverge from classical glucose exploitation, but activate glutamine synthesis, thereby serving this amino acid to cancer cells. Hence, due to the metabolic pressure applied by cancer cells, CAFs increase their incorporation of glucose-derived carbons into TCA metabolites and branched-chain amino acids-derived nitrogen to glutamine synthesis. Cancer cells educate CAFs to enhance their capability to use different nutrient sources to synthesize glutamine, in order to support cancer cell mitochondrial activity through glutaminolysis in stressed TME (86). A similar nutrient cross-talk mediated by exchanged glutamine has also been reported in models of astrocytes:glioblastoma and adipocytes:pancreatic cancer cells, as glutamine fuels the *de novo* purine biosynthesis (87, 88). Interestingly, glutamine within TME can also be active in rescheduling macrophages polarization toward the malignant M2 phenotype and enhancing cancer aggressiveness. Indeed, pharmacologic impairment of glutamine synthetase skews M2-polarized macrophages toward the M1-like phenotype. As a result of these metabolic changes M2 macrophages display a decreased ability to recruit immune and endothelial cells (89).

Beside glutamine, upon stromal autophagy activation, also alanine is largely secreted by pancreatic stellate cells, a stromal population very similar to activated CAFs. Alanine is uploaded by pancreatic cancer cells and fuels their TCA cycle over the glucose/glutamine-derived carbons, and this mitochondrial exploitation leads to an increased biosynthesis of lipids and non-essential amino acids (90). The metabolic rescheduling of pancreatic stroma has profound effects on cancer cells, as the contact with reactive stroma induces widespread histone acetylation in cancer cells, thereby serving to epigenetic purposes (91).

Moreover, mechanical signals sent by ECM composition and stiffness are able to reprogram CAFs and cancer cells toward a peculiar metabolic cross-talk mediated by exchange of aspartate and glutamate via the SLC1A3 transporter (92). The cross-talk is directional, as CAFs-derived aspartate feeds TCA cycle by sustaining the pyrimidine biosynthesis in cancer cells exposed to enhanced ECM stiffness, while glutamate provided by cancer cells is used by CAFs to maintain redox homeostasis through glutathione biosynthesis.

Finally, the tumor stroma cross-talk may affect kynurenine synthesis, a metabolite of tryptophan catabolism, through activation of the tryptophan 2,3-dioxygenase in CAFs. The shuttled kynurenine, uploaded by cancer cells, engages the EMT pathway, enhancing malignancy and immune suppression through regulation of dendritic and Th1 and Th2 subset of T cells (93).

#### Lipids

Lipids are surely key components of TME rescheduling of cancer cell metabolism. Indeed, lipids can be accumulated in cells, segregated to lipid droplets (LDs) due to physicochemical reasons, mainly as triglycerides and cholesterol derivatives. Their mobilization upon energetic request is under the control of specific lipases, tightly regulated by TME stimuli. Catabolism of triglycerides by adipose triglyceride lipase (ATGL) releases fatty acids (FAs), mainly used for energetic purposes via TCA cycle fueling, or for serving acetyl-CoA moieties for acetylation of proteins, either belonging to nuclear or cytosolic compartments. Cholesterol can be converted to 22- or 27-hydroxycholesterol, which activate liver X receptor signaling to up-regulate cholesterol efflux via regulation of the ATP-binding cassette transporters (94).

FAs, once released from LDs due to activation of ATGL, can be delivered to TME for fueling energetic needs of neighboring cells. To this end lipids can be loaded on secreted vesicles or translocated across the phospholipid bilayers of the plasma membrane through either passive diffusion or a protein-mediated transport system. Several membrane-associated FA binding proteins and transporters reportedly facilitate the transport process, including FA translocase (FAT, also named CD36), Fatty Acid Transport Protein and Plasma Membrane Fatty Acid Binding Protein. Highly aggressive prostate cancers show high expression of CD36 which facilitates the intake of exogenous FAs, and the subsequent LDs mobilization provokes a significant alteration in intracellular lipid content in terms of acyl-carnitines, monoacylglycerols and other lysophospholipids (95). Other findings have found the breast cancer cells resistant to HER2 therapy upregulate CD36, and thus acquiring an increased lipid metabolism and metabolic plasticity, both crucial for promoting resistant cells the adaptation and survival under nutrient deprivation and drug toxicity (96). Also, hypoxic breast and glioblastoma cells cancer cells upload FAs from the TME. The exploitation of triglycerides derived from accumulated LDs provides them ATP to face conditions of reoxygenation frequently occurring in a harsh TME (97). To note, stromal adipocytes are the main lipids donors in TME of several cancers. During melanoma progression adipocyte-derived lipids are taken up by FAT proteins, aberrantly expressed in melanoma, causing lipid upload and enhanced invasion and melanoma cell growth (98). The translocation of FAs in melanoma cells is also mediated by vesicles, as indicated by proteomic analysis of peritumoral adipocyte exosomes, rich in either lipids and enzymes involved in their catabolism (99). Moreover, in ovarian cancers, adipocytes promote tumor progression again through the provision of FAs. Although the exact mechanism through which adipocyte-derived FAs are transported into ovarian carcinoma cells remains uncertain, a role has been proposed for FAT/CD36 carrier (100). Besides adipocyte predominance in TME lipid supply, interestingly, levels of n-3 and n-6 polyunsaturated fatty acids (PUFA) and glycerophospholipids (e.g., phosphatidylcholine) have been highly detected in tumor cells cultured with endothelial cells (101). Collectively, these findings demonstrate that FATBPs and CD36 play a key role in tumor microenvironment metabolic cross-talk, driving the dependency of tumor cells toward exogenous lipid rewiring cancer cell metabolism and behavior. Furthermore, adipose-derived lipids have been also shown to mediate ovarian cancer chemoresistance (102). Indeed, a lipidomic analysis revealed that arachidonic acid AA is the key chemo-protective lipid mediator, although it is not known if arachidonate activity is due to its uploading or if it acts as a signaling molecule, as its sister companions prostaglandins. Finally, breast cancer cells promote lipolysis in peritumoral adipocytes leading to the release of FAs in the TME (103). Particularly, cancer cell-derived inflammatory signals induce an adipose triglyceride lipase-dependent catabolic pathway. The mobilized FAs, upon secretion, are transferred to cancer cells where they are stored in LDs or used through the carnitine palmitoyltransferase I-dependent fatty acid β-oxidation pathway, fueling a high mitochondrial activity.

#### Mitochondria

Nutrients are not only the unique metabolic molecules to be exchanges. Strikingly, horizontal transfer of intact and functional organelles (e.g., mitochondria) from stromal to cancer cells has been observed in TME. Cancer cells may exploit traveled mitochondria either to start or boost OXPHOS metabolism. Indeed, mitochondria-defective cancer cells *de novo* acquire mitochondria from TME to rescue a respiration they cannot carry out (104, 105). Oxidative stress is the driver for this mitochondrial transfer *via* cytoplasmic bridges (tunneling nanotubes) formed between bone marrow-derived MSCs and recipient leukemic blasts. The final outcome is an increase in mitochondrial mass, OXPHOS and ATP production as well as the drug resistance of cancer cells (106–108). Remarkably, CAFs channel their own mitochondria through intercellular interactions to further boost metabolism of OXPHOS-addicted prostate cancer cells. The molecular driver of such behavior seems to be again the lactate as its presence putatively enhances the formation of such mitochondria roads. These *de novo* achieved mitochondria are finely active for OXPHOS metabolism, ROS production and EMT promotion in cancer cells (51). Of note, these exchanges of intact mitochondria in prostate cancer also occur in xenografts of mice models and are not restricted to mitochondria-defective cancer cells.

#### Microvesicles

Extracellular vesicles (EVs) trafficking has been recently described as a new form of intercellular communication (87), with a high impact on the nutritional exchanges within tumor microenvironment either directly or indirectly through the cell-cell exchange of metabolic enzymes. EVs are approximately spherical structures limited by a lipid bilayer and containing bioactive components, such as proteins, lipids and nucleic acids. EVs are secreted by many cell populations, including fibroblasts (88), hematopoietic-derived cells, epithelial cells, neurons and tumor cells (109–111). EVs are classified into two main distinct subtypes, depending on their biogenesis, size, morphology and protein composition: exosomes and ectosomes/microvesicles (112). Exosomes are vesicles with a diameter of 50–150 nm, which are formed via inward budding of late endosomes membrane, the so called multivesicular bodies, which can fuse with the plasma membrane, releasing exosomes into the extracellular environment. On the other hand, microvesicles are directly produced by plasma membrane blebbing, are larger than exosomes, ranging from 100 nm to 1 μm in diameter (113). Once released into the extracellular environment they can interact to recipient cells receptors thereby triggering signal transduction events or they can fuse with the plasma-membrane of the acceptor cell releasing their content in the cytoplasm. EVs trafficking is involved in both physiological and pathological contexts such as: immunity (114), tissue regeneration (115), stem cell biology (116), angiogenesis (117), and tumor progression (118). Here we will focus our attention on EVs mediated-cross-talk in the context of tumor microenvironment. Tumor derived EVs are classically viewed as a way to alter tumor microenvironment to facilitate cancer progression via the transfer of proteins such as: (i) epidermal growth factor receptor-vIII, an oncogenic receptor (119); (ii) multidrug resistance-associated protein 1 a membrane protein mediating export of organic anions and drugs from the cytoplasm (120); (iii) pro-angiogenic proteins, i.e., TGF-β and VEGF, etc. (121). In addition, miRNA transferred by cancer EVs induce, by means of a still unknown mechanism, the secretion of CAFs chemokines such as CXCL1 and CXCL8 that correlate with poorer survival in gastric cancer patients (122).

More recently, it has become evident that CAFs are also able to produce and secrete EVs, thereby underlining the bi-directional importance of EVs trafficking within TME. Proteins and miRNA produced by CAFs and conveyed through EVs to tumor cells influence their behavior, supporting cancer cells growth rate and survival (123, 124), aggressiveness (125, 126) and favoring chemoresistance (127). In addition, CAF-derived EVs, uploaded by tumor cells, induce metabolic changes in acceptor cells such as enhanced glycolysis and glutamine metabolism rate, decreased oxygen consumption rate and down-regulation of mitochondrial function (128). The growing evidences about the involvement of EVs trafficking in regulating tumor cells metabolism has pushing the focus on the study of EV-transferred metabolites between different subsets of cells within tumor microenvironment. EVs-mediated trafficking of metabolites may be of particular importance for cancer cells that need a very high rate of metabolites influx to sustain their rapid cell growth. Currently, metabolomic studies on EVs has addressed their metabolic content in terms of lipids. It is reported that EVs transport plasma-membrane derived lipids i.e., sphingolipids, sterols, glycerophospholipids, fatty acids, and sphingolipids (129–131), with different relative proportion and composition reflecting those of their parental cells. Fewer studies are currently available describing the complete metabolome of the EVs, but what it is clearly emerging that, besides lipids, EVs contain many other organic molecules such as vitamins, amino acids, sugars, nucleotides, carnitines and aromatic compounds (128, 132, 133).

However, a very efficient way to induce a change in recipient cells phenotype, with respect to the simple transport of metabolites, is the transfer of enzymes involved in cellular metabolism. The analysis of the “vesiclepedia” database using informatics tools that clusterize the proteins contained into the EVs using a functional criterion, reveals that over 25% of them are directly involved in cell metabolism (113). In this view, EVs trafficking can be seen also as a metabolic coordination platform between the different cell populations within the solid tumor. This “metabolic synchronization” allows the optimization of the overall request for metabolites between the various cellular components of the tumor in order to support the survival and the neoplastic expansion of the tissue.

## Summary

Tumor-stroma metabolic cross-talk mainly portrays the setting where tumor confiscates metabolic nutrients, including lactate, amino acids and fatty acids, from local and/or stromal sources. This event provokes the catabolic pathways, such as autophagy, glycolysis and lipolysis in the tumor-associated cellular compartment. This interplay is absolutely reciprocal, as the interactions between stromal and tumor cells mutually reprogram the metabolism of each cell population. Highly aggressive cancer cells experience specific metabolic reprogramming, aimed at optimizing and functionally exploiting stromal cues (i.e., metabolites, vesicles, organelles), likely representing the critical transducers of the rewiring of the cancer metabolism within the TME. Stromal-induced mitochondrial dysregulation, in terms of oncometabolites production, ROS production and organelle biogenesis or transfer, contributes to the proliferative and metastatic potential of neoplastic cells. The flexibility that metabolic deregulation of cancer cells upon education by TME, often referred as metabolic plasticity, provides tumor cells the correct tools to face environmental hostile conditions.

Few therapeutic approaches have been developed to target tumor:stroma:metabolic interplay, among these we can cite glycolytic inhibitors to target the stromal component or pseudohypoxic cancer cells, or mitochondrial inhibitors for targeting mitochondrial metabolism in OXPHOS-addicted populations (134). The main obstacle in such targeting is the metabolic plasticity arising in this stroma-cancer communication: any strategy targeting one side of the tandem rapidly results in rescue of the other part of the tandem, to shift metabolism toward adaptation to dynamic environment. Although repurposing efforts to develop new metabolic inhibitors for cancer therapy to implement treatments is highly warranted, the preliminary strongest effort needed right now is the identification of the molecular player of metabolic plasticity, in order to efficiently target the adaptive symbiosis of tumor: stroma tandem.

## Author Contributions

PCh, PCi, LI, and GC contributed to the writing of this manuscript. Figure was rendered by LI. Editing was performed by all authors of this paper.

### Conflict of Interest

The authors declare that the research was conducted in the absence of any commercial or financial relationships that could be construed as a potential conflict of interest.

## Abbreviations

α-KG: α-ketoglutarate
CAAs: Cancer Associate Adipocytes
CAFs: cancer associated fibroblasts
ECM: extracellular matrix
CSF: colony stimulating factor
CXCL: chemokine (C-X-C motif) ligand
EMT: epithelial-to-mesenchymal transition
EVs: extracellular vesicles
FAs: fatty acids
FAO: fatty acid oxidation
HIF-1-α: hypoxia-inducible factor 1-α
IFN: interferon
IL: interleukin
LDs: lipid droplets
MCT: monocarboxylate transporter
MSCs: mesenchymal stem cells
OXPHOS: oxidative phosphorylation
PDGF: platelet derived growth factor
SDF-1: Stromal cell-Derived Factor-1
SLCs: solute carriers
TAMs: tumor associated macrophages
TGF-β: transforming growth factor-β
TCA: tricarboxylic acid cycle
TME: tumor microenvironment
VEGF: vascular endothelial growth factor
VEGF: vascular endothelial growth factor receptor.
